# Genomic profiling of colorectal cancer in large-scale Chinese patients: amplification and somatic mutations in ERBB2

**DOI:** 10.32604/or.2024.047309

**Published:** 2024-08-23

**Authors:** YUZHI LIU, EVELYNE BISCHOF, ZHIQIN CHEN, JIAHUAN ZHOU, BEI ZHANG, DING ZHANG, YONG GAO, MING QUAN

**Affiliations:** 1 Department of Oncology, Shanghai East Hospital, Tongji University School of Medicine, Shanghai, China; 2 State Key Laboratory of Oncogenes and Related Genes, Shanghai Cancer Institute, Department of Oncology, Renji Hospital, School of Medicine, Shanghai Jiao Tong University, Shanghai, China; 3 Jiangsu Hengrui Pharmaceuticals Co., Ltd., Shanghai, China; 4 3D Medicines Inc., Shanghai, China

**Keywords:** Colorectal cancer, ERBB2, HER2, Human epidermal growth factor receptor 2, Genetic profiling, Precision oncology

## Abstract

**Objectives:**

Human epidermal growth factor receptor 2 (HER2)-targeted therapies have demonstrated potential benefits for metastatic colorectal cancer (mCRC) patients with HER2 amplification, but are not satisfactory in cases of HER2 mutant CRCs.

**Methods:**

Consequently, further elucidation of amplifications and somatic mutations in erythroblastic oncogene B-2 (ERBB2) is imperative. Comprehensive genomic profiling was conducted on 2454 Chinese CRC cases to evaluate genomic alterations in 733 cancer-related genes, tumor mutational burden, microsatellite instability, and programmed death ligand 1 (PD-L1) expression.

**Results:**

Among 2454 CRC patients, 85 cases (3.46%) exhibited ERBB2 amplification, and 55 cases (2.24%) carried ERBB2 mutation. p.R678Q (28%), p.V8421 (24%), and p.S310F/Y (12%) were the most prevalent of the 16 detected mutation sites. In comparison to the ERBB2 altered (alt) group, KRAS/BRAF mutations were more prevalent in ERBB2 wild-type (wt) samples (ERBB2wt *vs*. ERBB2alt, KRAS: 50.9% *vs*. 25.6%, *p* < 0.05; BRAF: 8.5% *vs*. 2.3%, *p* < 0.05). 32.7% (18/55) of CRCs with ERBB2 mutation exhibited microsatellite instability high (MSI-H), while no cases with HER2 amplification displayed MSI-H. Mutant genes varied between ERBB2 copy number variation (CNV) and ERBB2 single nucleotide variant (SNV); TP53 alterations tended to co-occur with ERBB2 amplification (92.3%) as opposed to ERBB2 mutation (58.3%). KRAS and PIK3CA alterations were more prevalent in ERBB2 SNV cases (KRAS/PIK3CA: 45.8%/31.2%) compared to ERBB2 amplification cases (KRAS/PIK3CA: 14.1%/7.7%).

**Conclusion:**

Our study delineates the landscape of HER2 alterations in a large-scale cohort of CRC patients from China. These findings enhance our understanding of the molecular features of Chinese CRC patients and offer valuable implications for further investigation.

## Introduction

Colorectal cancer (CRC) is one of the most prevalent gastrointestinal malignancies globally, holding the third-highest incidence and the second-highest tumor-related mortality rates [1–3]. In China, CRC ranks as the third most diagnosed cancer and the fifth leading cause of cancer-related deaths [4–7]. Despite notable progress in targeted therapeutics, the 5-year survival rate for individuals battling metastatic or advanced colorectal cancer remains dismally low, hovering around 14% [8]. Colorectal cancer poses a significant public health challenge and comprehensive precision oncology approaches, including genetic landscape characterization of cancer, offer potential treatment targets and are crucial to improving early detection methods, enhancing therapeutic outcomes, and ultimately raising the survival rates for CRC patients [9–13].

Human epidermal growth factor receptor 2 (HER2) encoded by the erythroblastic oncogene B-2 (ERBB2) gene is a transmembrane tyrosine kinase receptor protein [14,15]. Heterodimerization of HER2 with other epidermal growth factor family members initiates its downstream signaling cascade, leading to cell proliferation and tumorigenesis [16]. In recent years, HER2-targeted therapies have been reported as beneficial in HER2-positive metastatic colorectal cancer (mCRC). While the frequencies of HER2 alterations (gene amplification or mutations) in CRC in Western populations are relatively well described (approximately 5%) in large-scale research data, such reporting on the HER2 status in Chinese CRC patients remains limited [17], with literature numbers varying from 2.6% to 11.2% [18–21]. Given the less satisfactory clinical response to HER2-targeted therapy in HER2 mutant CRCs compared to those with HER2 amplification, there is a need for a deeper understanding of amplification and somatic mutations in ERBB2 [16]. Particularly, exploring the correlation between HER2 status and other oncogenes is essential for comprehensive elucidation [22].

In the current study, we aimed to comprehensively describe the HER2 profile of Chinese CRC patients. We investigated a large cohort of 2454 CRC patients of Chinese origin, with a focus on the HER2 status via next-generation sequencing (NGS) analysis. We specifically conducted a deep dive into the clinical characteristics and genomic features of the CRC with ERBB2 alterations to draw conclusions and strategic therapeutic considerations. Our study contributes to enhancing the comprehension of molecular characteristics specific to Chinese CRC patients, offering valuable cues for subsequent investigations on practical applications in triage, decision-making, and prognostics. The current study delves into the intricate landscape of ERBB2 alterations, encompassing amplifications and sequence variants in CRC.

## Materials and Methods

### Patients

A total of 2454 Chinese patients with pathologically confirmed CRC diagnosis were enrolled, Samples were sequenced and analyzed in the 3Dmed Lab (Shanghai, China) between January 2017 and June 2020. The study was approved by the Ethics Committee of Shanghai East Hospital, Tongji University School of Medicine (No. 2020-073). All participants provided written informed consent.

### Tissue processing, DNA extractions, targeted sequencing and data processing

The sample processing and sequencing methods have been applied as previously described in detail [23], with small modifications. In brief, genomic DNA was extracted using the ReliaPrepTM FFPE gDNA Miniprep System (Promega, Madison, WI, USA) and quantified using the QubitTM dsDNA HS Assay Kit (Thermo Fisher Scientific, CA, USA). The next generation sequencing (NGS) library was constructed using the KAPA Hyper Prep Kit (KAPA Biosystems, Wilmington, MA, USA) and target capture was subjected to probe-based hybridization with a customized NGS panel, targeting 381 or 733 cancer-related genes.

Sequencing of captured libraries were analyzed using NovaSeq 6000 platform (Illumina, San Diego, CA, USA) with 100-bp paired-end reads and a mean coverage depth of 500×. Raw data of samples were aligned with the reference genome hg19 via the Burrows-Wheeler Aligner (v0.7.12). PCR duplicate reads were removed and sequence metrics were collected using Picard (v1.130) and SAMtools (v1.1.19), respectively. Variant calling was applied only in the targeted regions. An in-house developed R package was used to execute a variant detection model based on binomial tests to detect somatic single nucleotide variants (SNVs). Indels were detected through local realignment. Subsequently, variants were filtered based on the supporting read depth, strand bias, and base quality as described earlier [24]. An automated false positive filtering pipeline was then applied to all variants to ensure sensitivity and specificity at an allele frequency (AF) of ≥1%. Single-nucleotide polymorphisms (SNPs) and indels were annotated via ANNOVAR against the following databases: dbSNP (v138) (https://www.ncbi.nlm.nih.gov/snp/), ESP6500 (population frequency >0.015) (https://annovar.openbioinformatics.org/en/latest/user-guide/download/), and 1000 Genomes (https://www.ncbi.nlm.nih.gov/variation/tools/1000genomes/). Only missense, stop-gain, frameshift and non-frameshift indel mutations were kept. Copy number variations (CNVs) and gene rearrangements were detected as described previously [24]. Because of China human genetic resources law and patient privacy requirements, the sequence data were not publicly available. However, they were available upon request from the corresponding authors for the study purpose.

### Tumor mutational burden (TMB) analysis

TMB was defined as the number of somatic mutations in coding regions per megabase (muts/Mb) of genome examined. For better accuracy, two panels were used in this study, with the same method of TMB calculation. In order to increase the specificity, the mutations counted involved both synonymous and nonsynonymous mutations, as well as splicing variants, stop-gain and stop-loss. Indel variants involved both frameshift or non-frameshift insertions and deletions. Noncoding alterations were excluded.

### Microsatellite instability (MSI) analysis

The MSI analysis method has been described previously [25]. An in-house developed R script was used to assess the distribution of read counts among various repeat lengths for each microsatellite locus in each sample. An MSI score was determined as the percentage of unstable loci. Any sample with an MSI score of ≥0.4 was classified as MSI-high, and otherwise as microsatellite stability (MSS).

### Programmed cell death-ligand 1 (PD-L1) expression by immunohistochemistry (IHC)

FFPE tissue sections were subjected to assessment of PD-L1 expression using the PD-L1 IHC 22C3 pharmDx assay (Agilent Technologies, China).

### Statistical analysis

All the analyses were performed by GraphPad Prism 8.0 (GraphPad Software, USA) and R software (version 3.6.0, https://cran.r-project.org). The data were analyzed using *t*-test or one-way ANOVA to determine the differences between two groups; Fisher’s exact test was adopted to identify the association of two categorical variables. *p* values were two-tailed, and significance was defined as *p* < 0.05.

## Results

### Characteristics of the study population

In the present study, tumor tissues from a total of 2454 CRC patients with matched blood samples were sequenced and analyzed. As presented in Table 1, 60.2% were male patients, and the median age was 58 years (range, 17–90 years), the proportion of young CRC patients (defined as <50 years of age) was 26.9%. Of 2454 patients, 6.8% were confirmed to be with microsatellite instability (MSI)-high status, and 44.4% were programmed death ligand 1 (PD-L1) negative. The median tumor mutation burden (TMB) level was 7.26 muts/Mb. A total of 1975 (80.5%) tissue samples originated from the primary colon and rectum, and the remaining samples represented distant metastases obtained from liver (9.9%), lung (2.9) and other sites (6.8%).

**Table 1:** Clinicopathologic features in CRCs

Characteristics	Total (%)
**No. of patients**	2454
**Age**	
Median [Min, Max]	58.0 [17.0, 90.0]
<50	661 (26.9%)
>=50	1793 (73.1%)
**Sex**	
Male	1478 (60.2%)
Female	976 (39.8%)
**MSI status**	
MSI-H	168 (6.8%)
MSS	2239 (91.2%)
Missing	47 (1.9%)
**TMB level**	
Median [Min, Max]	7.26 [0, 600]
**PD-L1 expression**	
Negative	1090 (44.4%)
Positive	207 (8.4%)
Missing	1157 (47.1%)
**Sample location**	
Colon/Rectum	1975 (80.5%)
Liver	242 (9.9%)
Lung	71 (2.9%)
Other	166 (6.8%)

Abbreviation: CRC, colorectal cancer; MSI, microsatellite instability; MSS, microsatellite stability; TMB, tumor mutational burden; PD-L1, programmed cell death-ligand 1.

### Molecular landscape of somatic genomic alterations in Chinese CRC patients

**Figure 1:**
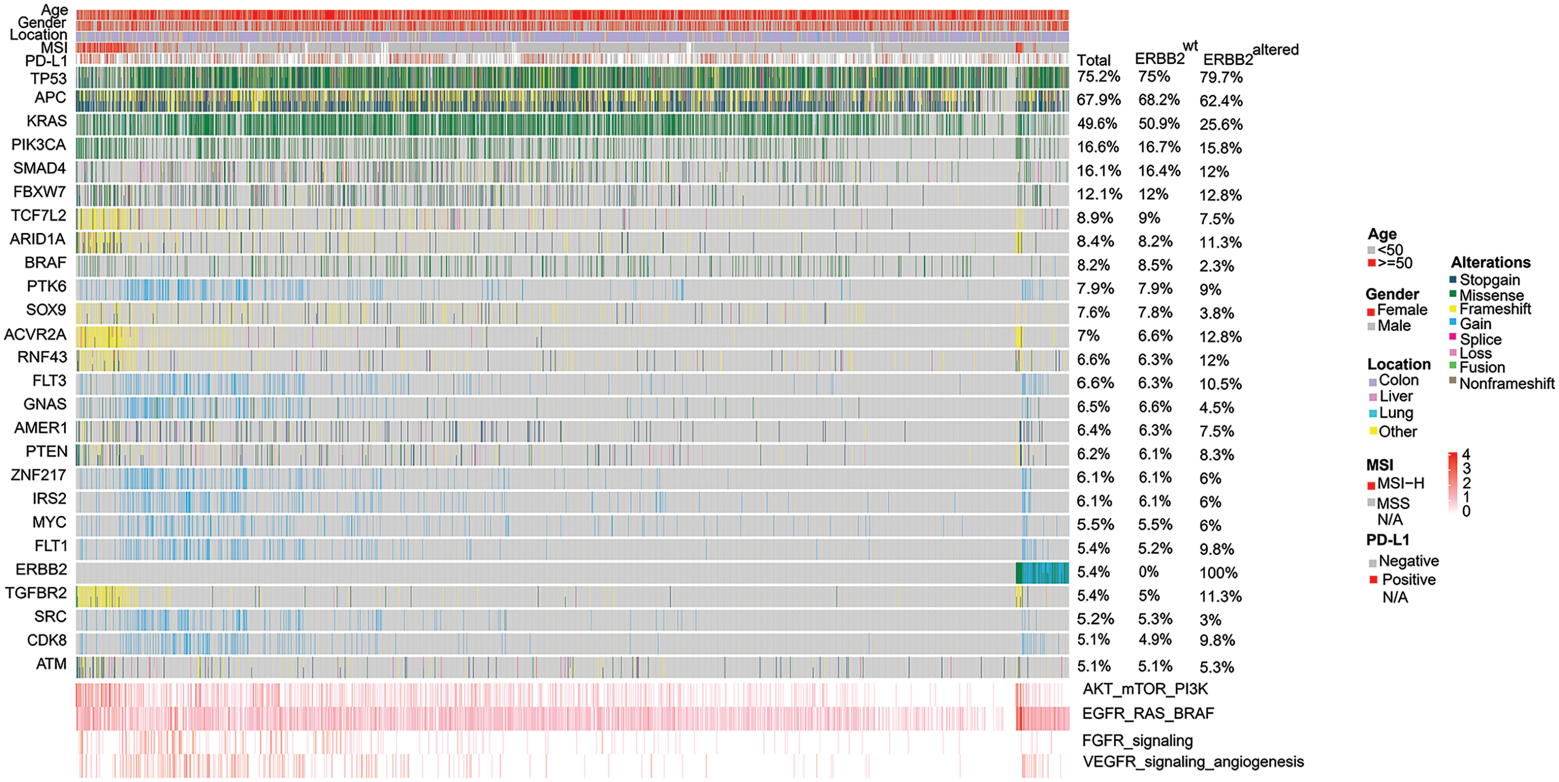
Heat graph of the common prevalent genomic changes and signaling pathways in 2454 Chinese colorectal cancer (CRC) patients. Genomic alterations involve stop-gain, missense, fusion, splice, frameshift, non-frameshift, and copy number variations (CNV) gain or loss. Alterations in the EGFR/RAS/BRAF pathway was observed most frequently, followed by AKT/mTOR/PI3K activation, VEGFR signaling mediated-angiogenesis and FGFR signaling pathway alterations.

Gene mutations in TP53 (75.2%), APC (67.9%) and KRAS (49.6%) were observed mostly, followed by PIK3CA (16.6%), SMAD4 (16.1%), FBXW7 (12.1%), TCF7L2 (8.9%), ARID1A (8.4%), BRAF (8.2%). The prevalence of ERBB2 alteration was 5.4% (Fig. 1). The key signaling pathways alteration was seen in the EGFR/RAS/BRAF signaling pathway (74.4% (n = 1826) of CRC patients), followed by AKT/mTOR (39.2%, n = 963) and VEGFR (16.6%, n = 408) signaling pathways. The mutational frequencies in total, ERBB2 wild-type and ERBB2 altered samples were listed separately, with no analysis of the germline mutations.

### Landscape and analysis of amplification and short variant mutations in ERBB2

**Figure 2:**
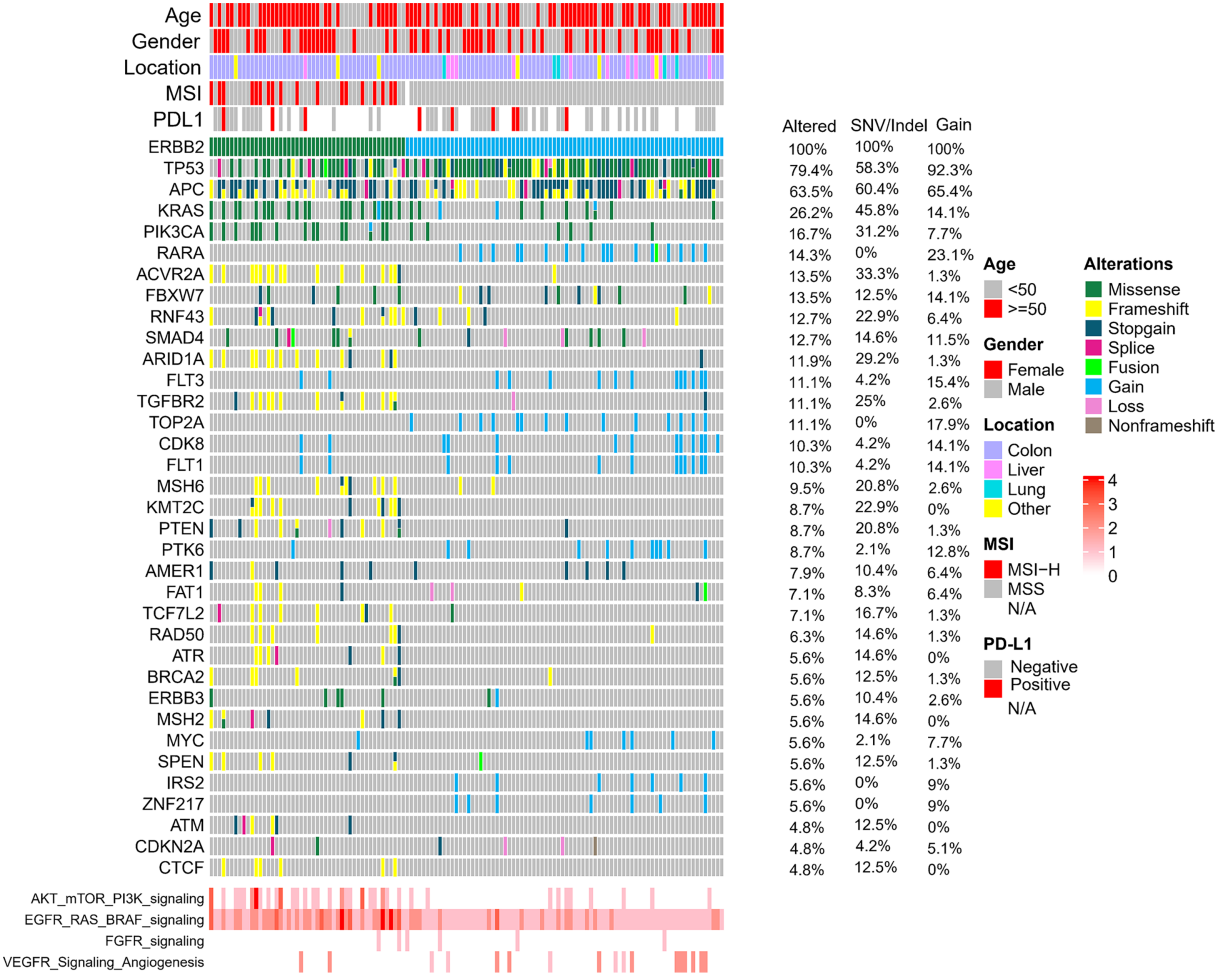
Overview of the common genomic changes and signaling pathways in CRC patients with ERBB2 alterations. The mutational frequencies in 133 ERBB2-altered, 85 ERBB2-amplified and 55 ERBB2-mutated samples are represented.

The relative frequencies of ERBB2 amplification and sequence variants were assessed. A total of 133 samples (5.4%) expressed alterations affecting ERBB2: ERBB2 amplification (85 cases; 3.46% of 2454 patients), ERBB2 sequence variants (SNP/Indel) (55 cases; 2.24% of 2454 patients), cooccurring SNP/Indel and ERBB2 amplification alterations (Fig. 2). The ERBB2 alterations were not significantly associated with clinicopathological characteristics such as sex, tumor locations, or PD-L1expression level. Consistent with previous studies, we observed that among the HER2-mutated cases, 32.7% were MSI-H, whereas none of the HER2 amplification cases were MSI-H [26].

**Figure 3:**
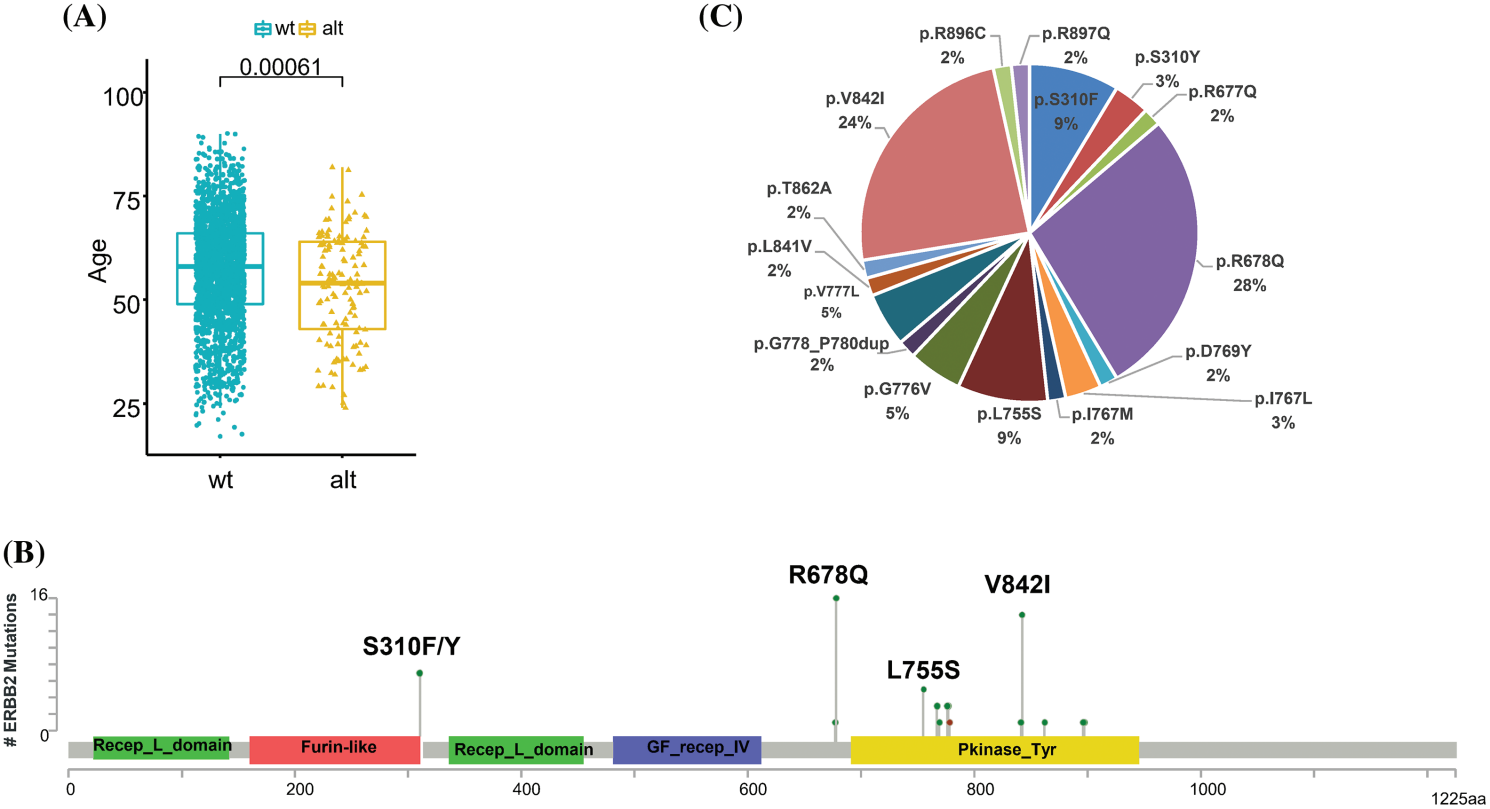
Comprehensive analysis of ERBB2 in CRC. (A) Relationship between HER2 status and age. (B) The frequency of ERBB2 mutational type in 55 ERBB2-mutated CRC samples. (C) Schematic diagram of domains and mutation sites of ERBB2 gene in CRC patients.

Interestingly, ERBB2 alterations were more likely to occur in younger CRCs (*p* = 0.00061, Fig. 3A). Of the 85 samples with amplification of ERBB2, the median copy number was 30, ranging from 4 to 245. In the analysis of ERBB2 mutation sites, 16 somatic ERBB2 SNVs/indels were identified in 55 patients. The most frequent alterations occurred on exon 17, p.R 678Q (28%), followed by exon 21, p.V842I (24%) and p.S310F/Y (12%) (Figs. 3B and 3C).

**Figure 4:**
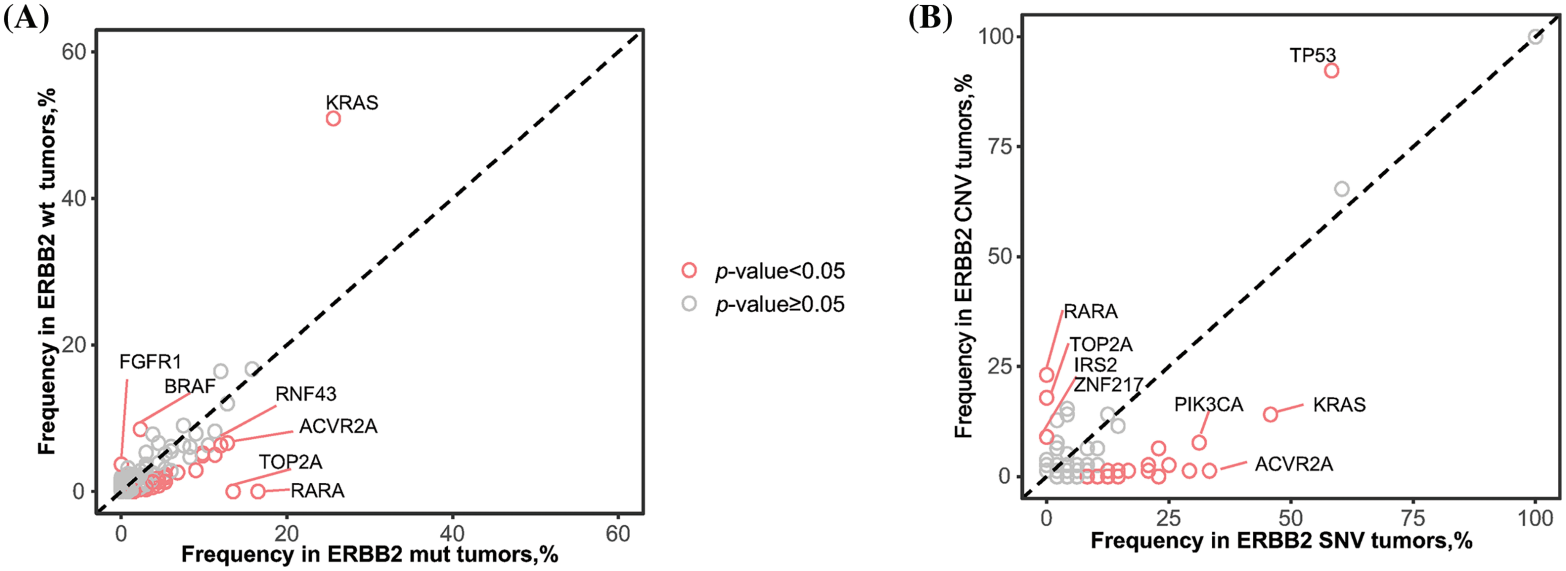
Comparison of alternative driver genes depended on the HER2 status. (A) Differences of frequency of alternative driver genes between ERBB2 wild-type (wt) and ERBB2 alteration (alt) samples. (B) Differences of frequency of alternative driver genes between ERBB2 copy number variations (CNV) and ERBB2 somatic single nucleotide variant (SNV). Each dot represents one gene, red dots indicates statistical significance.

KRAS mutations were more frequent in ERBB2 wild-type (ERBB2wt) samples than those with ERBB2 alterations (50.9% *vs*. 25.6%). Furthermore, BRAF mutations occurred at a frequency of 8.5% in ERBB2wt samples, while in ERBB2 alteration samples, the frequency of BRAF mutations was significantly lower at 2.3%. Conversely, RNF43 mutations exhibited an increased frequency in ERBB2 alteration samples (12.0%) as opposed to ERBB2wt samples (6.3%). Mutation frequencies of various genes, including RARA, TOP2A, and ACVR2A, showed significant differences between ERBB2wt and alteration samples (Figs. 1 and 4A).

Additionally, we conducted a comparison of mutant genes between patients exhibiting ERBB2 copy number variation (CNV) and those with ERBB2 single nucleotide variation (SNV). TP53 alterations were significantly more likely to co-occur with ERBB2 amplification (92.3%) than with ERBB2 mutation (58.3%). Conversely, KRAS alterations were notably more prevalent in ERBB2 SNV samples (45.8%) compared to ERBB2 amplification samples (14.1%). Similar trends were observed for PIK3CA (CNV: 7.7% *vs*. SNV: 31.2%) (Figs. 2 and 4B).

## Discussion

For the treatment of colorectal cancer Precision oncology has become increasingly important. HER2 is a promising target of mCRC, there have been several clinical trials conducted in HER2-positive mCRCs, and have shown encouraging results. CRC is one of the most common cancer types in China. Previous molecular profile studies mostly focused on the HER2 overexpression or gene amplification in Chinese CRC population [21,27]. Herein, we performed a comprehensive genome prefilling in a large sample of Chinese CRC patients, with a focus on ERBB2 amplification and somatic mutation analysis. Our findings provide a thorough comprehension of the relative frequencies and characteristics associated with these ERBB2 alterations, illuminating their potential implications in CRC.

### Landscape of HER2 alteration in Chinese CRC patients

In the current study, the incidence of ERBB2 alteration was 5.4%, consistent with published data [17]. Among these 2454 patients, 3.46% carried ERBB2 amplification, and 2.24% carried ERBB2 mutation. In the reported data in Western populations (cBioPortal and MDACC), ERBB2 mutations were most commonly observed in the tyrosine kinase domain (46%), with mutations in exon 20 (20%), exon 19 (11%), and exon 21 (9%) across all cancers [28]. Additionally, 37% of HER2 mutations are reported in the extra-cellular domain. In colorectal cancer, the most prevalent HER2 mutations occurred in exon 21 (23%) and the extracellular domain (23%), while the V842I variant in exon 21 was most common (19%) [28]. In the current study on almost 3000 Chinese subjects, R678Q in the transmembrane domain was the most frequently observed HER2 mutation, followed by V842I in exon 21 and S310F/Y in the extracellular domain (Table 2). Knowing the clinical significance of the common and unique genomic changes among the Chinese and Western populations would be intriguing.

**Table 2:** ERBB2 mutation hotspots vary between Chinese and Western population

Chinese population			Western population		
Frequency	Specific variant	Location	Frequency	Specific variant	Location
28%	R678Q	Transmembrane	18.7%	V842I	Exon 21
24%	V842I	Exon 21	6.9%	V777L	Exon 20
9%	S310F	Extra-cellular	5.9%	R678Q	Transmembrane
9%	L755S	Exon 19	4.1%	S310F	Extra-cellular
5%	V777L	Exon 20	3.2%	L755S	Exon 19

### HER2-targeted therapies in mCRC

Previous clinical studies have shown that HER2 targeted therapy might become a potential treatment option for HER2-overexpressed/amplified mCRC in the late-line setting [29–33]. For instance, MyPathway, a basket study investigated the efficacy and safety of pertuzumab and trastuzumab in solid tumors with HER-2-positive. A total of 57 patients with mCRC were enrolled, achieving an objective response rate (ORR) of 32%, and the median progression-free survival (PFS) was 2.9 months. Notably, subgroup analysis revealed that HER-2-positive and KRASwt patients had a longer median PFS (5.3 months) and median overall survival (mOS) (17 months), compared with KRAS mutant patients (mPFS: 1.4 months, mOS: 7 months), which indicated that patients cooccurring with HER2 amplification and KRAS mutation may have limited benefit from HER2-targeted therapy [29]. The combination of trastuzumab and lapatinib in the HERACLES-A study, resulted in an ORR of 30%, with a median PFS of 21 weeks and a median OS of 46 weeks in patients with KRASwt and HER2-positive mCRC [32]. Based on the previous study, HERACLES-B further explored the efficacy and safety of pertuzumab in combination with HER2-antibody-drug conjugate (ADC), trastuzumab-entansine (T-DM1) for RAS/BRAF wild- type and HER-2-positive colorectal cancer, while the ORR was only 9.7% with a median PFS of 4.1 months [33].

However, a novel HER2-ADC, T-DXd displayed a promising therapeutic actionability in patients with RAS/BRAF wild-type and HER2-positive mCRC, achieving an ORR of 45.3% (24/53), with a median PFS of 6.9 months. But unlike the T-DXd in patients with HER2-low breast cancer, patients with HER2 2+ or HER2 1+ mCRC had no confirmed objective response to T-DXd [34]. Moreover, in the MOUNTAINEER study, trastuzumab plus tucaninib (an anti-HER2 tyrosine kinase inhibitor) showed an impressive result that the ORR was 38.1% with a median PFS of 8.2 months, and a median OS of 24.1 months [35]. These findings have suggested that dual-target anti-HER2 treatment and HER2-ADC drugs might be a potential anti-HER2 strategy in HER2-overexpressed/amplified mCRC.

Several ERBB2 mutation sites showed sensitivity to HER2-targeted therapies in preclinical studies [36], however few articles explored the therapeutic options for mCRC patients with ERBB2 mutations. The SUMMIT [37] and MyPathway [29] basket trials showed prospective clinical data on the efficacy of HER2-targeted treatment in ERBB2 mutated mCRCs. In the SUMMIT trial, neratinib was administered to 125 patients with ERBB2 mutated advanced solid tumor, of which 12 were diagnosed with mCRC. Differently from the good activity in breast cancer, neratinib did not elicit any response in mCRC patients, indicating that the efficacy of single-agent pan-HER kinase inhibition may vary depending on the histology in this tumor type. Of note, two of three mCRC patients with V842I mutation achieved stable disease as best response [37]. In the MyPathway trial on pertuzumab and trastuzumab, three of 57 patients with ERBB2 amplified mCRCs harbored concurrent ERBB2 mutations. Of these, one had partial response as best response [29]. In addition, another case report presented a mCRC patient with ERBB2 mutation (L755S) and concurrent APC and BRAF alterations receiving a combination of 5-Fu and leucovorin plus trastuzumab, but progressed after only 6 weeks [38].

Here, we observed that the molecular features between ERBB2 amplification and ERBB2 mutation are distinct. For instance, TP53 alterations were more prevalent in cases with ERBB2 amplification (92.3%) *vs*. ERBB2 mutation (58.3%), while the incidence of KRAS positive in ERBB2 SNV samples reached up to 45.8%, higher than that in those with ERBB2 amplification (14.1%), so did the PIK3CA (CNV: 7.7% *vs*. SNV: 31.2%). The impact of these specific differences and mechanism of HER2 mutations on the effect of HER2-targeted treatments for CRC requires further investigation.

### Relationship between HER2 alteration and clinical features, genomic changes and signaling pathways in CRC

We further assessed the correlations of HER2 alteration with clinicopathological features in CRC. Multiple studies have reported a connection between HER2 amplification and tumor location, nerve invasion, peritoneal metastasis and Duke’s stage [17,28,39,40]. A retrospective study conducted in Eastern China revealed that HER2 amplification was related to more extensive invasion of intestinal wall and higher TNM stage in CRC patients with stages I–III [21], while other reports did not find such association [27,41]. The HER2 status had no significant correlation with gender, tumor locations, or the expression level of PD-L1 in our cohort. However, we found that ERBB2 alterations were more prone to occur in younger patients with CRCs, which is an important finding given that incidence of younger patients with colorectal cancer have been increasing steadily [42].

The KRAS and BRAF status holds pivotal significance in tailoring treatment strategies and predicting the prognosis of colorectal cancer (CRC). Extensive investigations have explored the correlation between these oncogenes and HER2 in CRC. Prior studies have demonstrated a mutually exclusive relationship between KRAS mutations and HER2 amplification [43]. In a meta-analysis of 3256 patients with mCRC, HER2 amplification was associated with KRAS/BRAF wild-type status regardless of disease stage [44]. Similarly, in the PETACC-8 study, HER2 alterations were predominant in patients with KRASwt tumors compared with those with KRAS mutations (5.6% *vs*. 2.4%, *p* < 0.001) [45]. In Chinese population, a study of 139 CRC patients has shown that there is no relationship between HER2 amplification and KRAS status (*p* = 0.052), while the odds ratio was very low (0.279) [46]. Herein, we found that KRAS mutations or BRAF mutations occurred more frequently in ERBB2wt samples, as compared with ERBB2 alteration ones (ERBB2wt *vs*. ERBB2 alteration, KRAS: 50.9% *vs*. 25.6%, *p* < 0.05; BRAF: 8.5% *vs*. 2.3%, *p* < 0.05). Our findings suggest an exclusive relationship between KRAS/BRAF mutation and HER2 status.

MSI-H serves as a biomarker for the efficacy of immune checkpoint inhibitors in patients with mCRC. A retrospective study of 731 Chines CRC patients found that 48.3% (14/29) of cases with HER2 mutation were MSI-H, while no cases with HER2 amplification had MSI-H [26], such findings were also presented in our study, and the incidence of MSI-H in HER2 mutated cases was 32.7%. In the Western population, it has also been reported that there is a negative relationship between HER2 amplification and MSI-H [47]. Furthermore, in the small-sample (n = 25) MSI-H subgroup of previous study [26], patients with HER2 mutation had a significantly worse median progression free survival for anti-PD-1 treatment than those without HER2 alteration (*p* = 0.036). However, the internal relationship between HER2 mutation and MSI-H and the role of HER2 alterations in immunotherapy requires further study.

Our study further delved into the co-occurrence of ERBB2 alterations with mutations in other critical genes in CRC. The frequency of KRAS mutations was significantly higher in ERBB2 wild-type (ERBB2wt) samples, unveiling a potential interplay between KRAS and ERBB2 alterations. Conversely, BRAF mutations were more frequent in ERBB2wt samples, hinting at distinct mutational patterns associated with ERBB2 status. Additionally, RNF43 mutations exhibited an increased frequency in ERBB2 alteration samples, suggesting intricate genomic relationships that merit further exploration.

Comparative analysis between ERBB2 copy number variations (CNV) and single nucleotide variations (SNV) provided additional insights. TP53 alterations were notably enriched in ERBB2 amplification cases, implying potential co-occurrence patterns with TP53 mutations. Conversely, KRAS and PIK3CA mutations were more prevalent in ERBB2 SNV cases, indicating possible pathways of oncogenic cooperation distinct from CNV cases.

**Figure 5:**
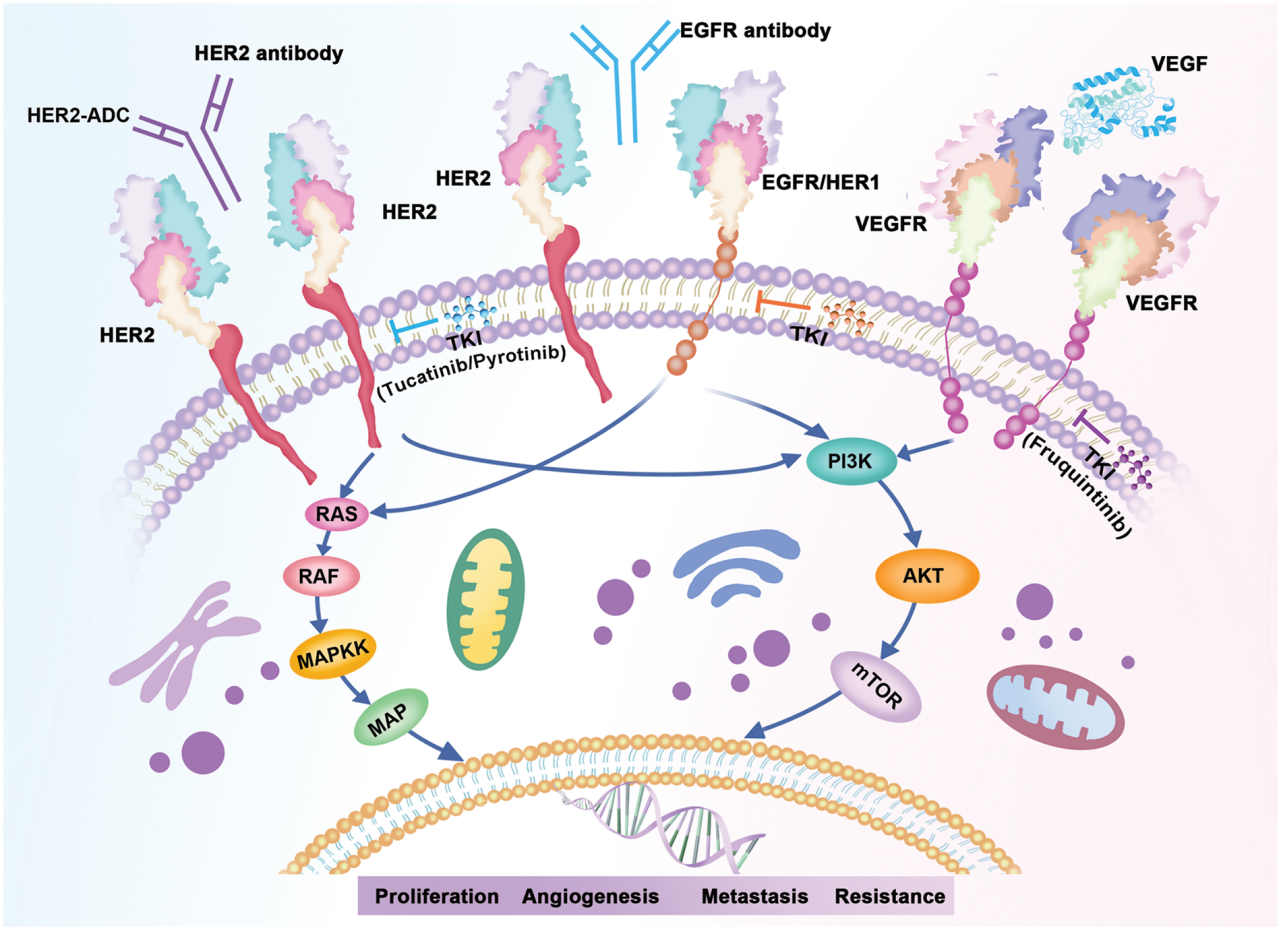
HER2 associated signaling pathways.

The overexpression of EGFR and HER2 has been reported to have significant impacts on the colorectal cancer (CRC) development and metastasis [48]. In the current study, we found that both in CRC patients and those with ERBB2 alterations, EGFR/RAS/BRAF pathway, AKT/mTOR/PI3K pathway, and VEGFR signaling pathway alterations were observed most frequently. As illustrated by Fig. 5, all these cellular signaling pathways were associated with HER2 causing tumor progression. Given that the three signaling pathways are the most important abnormal signaling pathways in colorectal cancer [49–51], combining HER2-targeted therapy with above pathway inhibitors may have potential efficacy in colorectal cancers with HER2 alterations.

The study had several limitations. Firstly, the HER2 alteration landscape was based on the NGS panel assay, though it contains most of the crucial genes in CRC, other potential genes may be overlooked. In addition, germline mutations were not involved in the analysis of HER2 alteration, thus comprehensive whole-genome profiling and integrated analysis of somatic and germline mutations need to be undertaken. Moreover, information on the treatment and prognosis matching genomics are limited, the implication of HER2 status on prognosis should be further explored by well-designed research in the future.

In this study, the use of comprehensive genomic profiling on this large cohort of Chinese CRC cases allowed us to describe the HER2 landscape of Chinese CRCs and better understand the relationship between HER2 status and other oncogenes. Since several HER2-targeted therapies have shown potential benefit for HER2-positive mCRC, HER2 alterations, especially ERBB2 mutation sites on the effect of current HER2-targeted treatments is worth further investigation.

### Conclusions

In summary, our comprehensive analysis of ERBB2 alterations in CRC unravels intriguing associations and highlights potential therapeutic avenues. Further investigations into the functional consequences of these alterations and their interplay with other critical mutations are warranted to advance our understanding of CRC biology and pave the way for targeted therapeutic strategies.

## Data Availability

The datasets generated during and/or analyzed during the current study are available from the corresponding author on request.

## Abbreviations

CNV: Copy number variation
CRC: Colorectal cancer
ERBB2: Erythroblastic oncogene B-2
HER2: Human epidermal growth factor receptor 2
IHC: Immunohistochemistry
MSI: Microsatellite instability
MSS: Microsatellite stability
NGS: Next generation sequencing
PD-L1: Programmed cell death-ligand 1
SNVs: Single nucleotide variant
TMB: Tumor mutational burden
wt: Wild-type

## References

[1] Sung, H., Ferlay, J., Siegel, R. L., Laversanne, M., Soerjomataram, I. et al. (2021). Global cancer statistics 2020: GLOBOCAN estimates of incidence and mortality worldwide for 36 cancers in 185 countries. CA: A Cancer Journal for Clinicians*,* 71 *(*3*),* 209–249; 33538338 10.3322/caac.21660

[2] Morgan, E., Arnold, M., Gini, A., Lorenzoni, V., Cabasag, C. J. et al. (2023). Global burden of colorectal cancer in 2020 and 2040: Incidence and mortality estimates from GLOBOCAN. Gut*,* 72 *(*2*),* 338–344. 10.1136/gutjnl-2022-327736; 36604116

[3] Xi, Y., Xu, P. (2021). Global colorectal cancer burden in 2020 and projections to 2040. Translational Oncology*,* 14 *(*10*),* 101174. 10.1016/j.tranon.2021.101174; 34243011 PMC8273208

[4] Qiu, H., Cao, S., Xu, R. (2021). Cancer incidence, mortality, and burden in China: A time-trend analysis and comparison with the United States and United Kingdom based on the global epidemiological data released in 2020. Cancer Communications*,* 41 *(*10*),* 1037–1048. 10.1002/cac2.v41.10 34288593 PMC8504144

[5] Yang, Y., Han, Z., Li, X., Huang, A., Shi, J. et al. (2020). Epidemiology and risk factors of colorectal cancer in China. Chinese Journal of Cancer Research*,* 32 *(*6*),* 729–741. 10.21147/j.issn.1000-9604.2020.06.06; 33446996 PMC7797231

[6] Shao, B., Zhu, M., Shen, K., Luo, L., Du, P. et al. (2023). Disease burden of total and early-onset colorectal cancer in China from 1990 to 2019 and predictions of cancer incidence and mortality. The Journal of Clinical Epidemiology*,* 15*,* 151–163. 10.2147/CLEP.S391058; 36755975 PMC9900241

[7] Qu, R., Ma, Y., Zhang, Z., Fu, W. (2022). Increasing burden of colorectal cancer in China. The Lancet Gastroenterology & Hepatology*,* 7 *(*8*),* 700. 10.1016/S2468-1253(22)00156-X; 35809603

[8] Siegel, R. L., Miller, K. D., Goding Sauer, A., Fedewa, S. A., Butterly, L. F. et al. (2020). Colorectal cancer statistics, 2020. CA: A Cancer Journal for Clinicians*,* 70 *(*3*),* 145–164; 32133645 10.3322/caac.21601

[9] di Nicolantonio, F., Vitiello, P. P., Marsoni, S., Siena, S., Tabernero, J. et al. (2021). Precision oncology in metastatic colorectal cancer—from biology to medicine. Nature Reviews Clinical Oncology*,* 18 *(*8*),* 506–525. 10.1038/s41571-021-00495-z; 33864051

[10] Jensen, L. H., Rogatto, S. R., Lindebjerg, J., Havelund, B., Abildgaard, C. et al. (2023). Precision medicine applied to metastatic colorectal cancer using tumor-derived organoids and in-vitro sensitivity testing: A phase 2, single-center, open-label, and non-comparative study. Journal of Experimental & Clinical Cancer Research*,* 42 *(*1*),* 115. 10.1186/s13046-023-02683-4; 37143108 PMC10161587

[11] Muller, C., Yurgelun, M., Kupfer, S. S. (2020). Precision treatment and prevention of colorectal cancer-hope or hype? Gastroenterology*,* 158 *(*2*),* 441–446. 10.1053/j.gastro.2019.09.046; 31622623 PMC6957699

[12] Lim, S. H., Cho, H. J., Kim, K. M., Lim, H. Y., Kang, W. K. et al. (2023). Comprehensive molecular analysis to predict the efficacy of chemotherapy containing bevacizumab in patients with metastatic colorectal cancer. Oncology Research*,* 31 *(*6*),* 855–866. 10.32604/or.2023.030374; 37744267 PMC10513961

[13] Guo, W., Cai, Y., Liu, X., Ji, Y., Zhang, C. et al. (2023). Single-exosome profiling identifies ITGB3+ and ITGAM+ exosome subpopulations as promising early diagnostic biomarkers and therapeutic targets for colorectal cancer. Research*,* 6*,* 0041. 10.34133/research.0041; 37040507 PMC10076010

[14] Iqbal, N., Iqbal, N. (2014). Human epidermal growth factor receptor 2 (HER2) in cancers: Overexpression and therapeutic implications. Molecular Biology International*,* 2014*,* 852748; 25276427 10.1155/2014/852748 PMC4170925

[15] Rubin, I., Yarden, Y. (2001). The basic biology of HER2. Annals of Oncology*,* 12 *(*Suppl 1*),* S3–S8; 11521719 10.1093/annonc/12.suppl_1.s3

[16] Bai, X., Sun, P., Wang, X., Long, C., Liao, S. et al. (2023). Structure and dynamics of the EGFR/HER2 heterodimer. Cell Discovery*,* 9 *(*1*),* 18. 10.1038/s41421-023-00523-5; 36781849 PMC9925823

[17] Siena, S., Sartore-Bianchi, A., Marsoni, S., Hurwitz, H. I., McCall, S. J. et al. (2018). Targeting the human epidermal growth factor receptor 2 (HER2) oncogene in colorectal cancer. Annals of Oncology*,* 29 *(*5*),* 1108–1119. 10.1093/annonc/mdy100; 29659677 PMC5961091

[18] Wang, X. Y., Zheng, Z. X., Sun, Y., Bai, Y. H., Shi, Y. F. et al. (2019). Significance of HER2 protein expression and HER2 gene amplification in colorectal adenocarcinomas. World Journal of Gastrointestinal Oncology*,* 11 *(*4*),* 335–347. 10.4251/wjgo.v11.i4.335; 31040898 PMC6475672

[19] Shan, L., Lv, Y., Bai, B., Huang, X., Zhu, H. (2018). Variability in HER2 expression between primary colorectal cancer and corresponding metastases. Journal of Cancer Research and Clinical Oncology*,* 144 *(*11*),* 2275–2281. 10.1007/s00432-018-2744-z; 30203148 PMC11813305

[20] Liu, F., Ren, C., Jin, Y., Xi, S., He, C. et al. (2020). Assessment of two different HER2 scoring systems and clinical relevance for colorectal cancer. Virchows Archiv*,* 476 *(*3*),* 391–398. 10.1007/s00428-019-02668-9; 31720832 PMC7085476

[21] Zhang, X., Wu, J., Wang, L., Zhao, H., Li, H. et al. (2020). HER2 and BRAF mutation in colorectal cancer patients: A retrospective study in Eastern China. PeerJ*,* 8*,* e8602. 10.7717/peerj.8602; 32095377 PMC7023828

[22] Djaballah, S. A., Daniel, F., Milani, A., Ricagno, G., Lonardi, S. (2022). HER2 in colorectal cancer: The long and winding road from negative predictive factor to positive actionable target. In: American Society of Clinical Oncology educational book*,* vol. 42*,* pp. 219–232. 10.1200/EDBK_351354 35580290

[23] Su, D., Zhang, D., Chen, K., Lu, J., Wu, J. et al. (2017). High performance of targeted next generation sequencing on variance detection in clinical tumor specimens in comparison with current conventional methods. Journal of Experimental & Clinical Cancer Research*,* 36 *(*1*),* 121. 10.1186/s13046-017-0591-4; 28882180 PMC5590190

[24] Zhang, X., Mao, T., Zhang, B., Xu, H., Cui, J. et al. (2022). Characterization of the genomic landscape in large-scale Chinese patients with pancreatic cancer. eBioMedicine*,* 77*,* 103897. 10.1016/j.ebiom.2022.103897; 35231699 PMC8886010

[25] Xiao, J., Li, W., Huang, Y., Huang, M., Li, S. et al. (2021). A next-generation sequencing-based strategy combining microsatellite instability and tumor mutation burden for comprehensive molecular diagnosis of advanced colorectal cancer. BMC Cancer*,* 21 *(*1*),* 282. 10.1186/s12885-021-07942-1; 33726687 PMC7962287

[26] Qiu, M. Z., He, C. Y., Yang, X. H., Yang, L. Q., Lin, J. Z. et al. (2021). Relationship of HER2 alteration and microsatellite instability status in colorectal adenocarcinoma. Oncologist*,* 26 *(*7*),* e1161–e1170. 10.1002/onco.13786; 33844372 PMC8265359

[27] Ni, S., Wang, X., Chang, J., Sun, H., Weng, W. et al. (2022). Human epidermal growth factor receptor 2 overexpression and amplification in patients with colorectal cancer: A large-scale retrospective study in Chinese population. Frontiers in Oncology*,* 12*,* 842787. 10.3389/fonc.2022.842787; 35574415 PMC9097912

[28] Robichaux, J. P., Elamin, Y. Y., Vijayan, R. S. K., Nilsson, M. B., Hu, L. et al. (2019). Pan-cancer landscape and analysis of ERBB2 mutations identifies poziotinib as a clinically active inhibitor and enhancer of T-DM1 activity. Cancer Cell International*,* 36 *(*4*),* 444–457. 10.1016/j.ccell.2019.09.001; 31588020 PMC6944069

[29] Meric-Bernstam, F., Hurwitz, H., Raghav, K. P. S., McWilliams, R. R., Fakih, M. et al. (2019). Pertuzumab plus trastuzumab for HER2-amplified metastatic colorectal cancer (MyPathway): An updated report from a multicentre, open-label, phase 2a, multiple basket study. The Lancet Oncology*,* 20 *(*4*),* 518–530. 10.1016/S1470-2045(18)30904-5; 30857956 PMC6781620

[30] Nakamura, Y., Okamoto, W., Kato, T., Hasegawa, H., Kato, K. et al. (2019). TRIUMPH: Primary efficacy of a phase II trial of trastuzumab (T) and pertuzumab (P) in patients (pts) with metastatic colorectal cancer (mCRC) with HER2 (ERBB2) amplification (amp) in tumour tissue or circulating tumour DNA (ctDNA): A GOZILA sub-study. Annals of Oncology*,* 30*,* v199–v200. 10.1093/annonc/mdz246.004

[31] Strickler, J. H., Zemla, T., Ou, F. S., Cercek, A., Wu, C. et al. (2019). Trastuzumab and tucatinib for the treatment of HER2 amplified metastatic colorectal cancer (mCRC): Initial results from the MOUNTAINEER trial. Annals of Oncology*,* 30 *(*Suppl 5*),* v200.

[32] Sartore-Bianchi, A., Trusolino, L., Martino, C., Bencardino, K., Lonardi, S. et al. (2016). Dual-targeted therapy with trastuzumab and lapatinib in treatment-refractory, KRAS codon 12/13 wild-type, HER2-positive metastatic colorectal cancer (HERACLES): A proof-of-concept, multicentre, open-label, phase 2 trial. The Lancet Oncology*,* 17 *(*6*),* 738–746. 10.1016/S1470-2045(16)00150-9; 27108243

[33] Sartore-Bianchi, A., Lonardi, S., Martino, C., Fenocchio, E., Tosi, F. et al. (2020). Pertuzumab and trastuzumab emtansine in patients with HER2-amplified metastatic colorectal cancer: The phase II HERACLES-B trial. ESMO Open*,* 5 *(*5*),* e000911. 10.1136/esmoopen-2020-000911; 32988996 PMC7523198

[34] Siena, S., di Bartolomeo, M., Raghav, K., Masuishi, T., Loupakis, F. et al. (2021). Trastuzumab deruxtecan (DS-8201) in patients with HER2-expressing metastatic colorectal cancer (DESTINY-CRC01): A multicentre, open-label, phase 2 trial. The Lancet Oncology*,* 22 *(*6*),* 779–789. 10.1016/S1470-2045(21)00086-3; 33961795

[35] Strickler, J. H., Cercek, A., Siena, S., André, T., Ng, K. et al. (2022). LBA27-additional analyses of MOUNTAINEER: A phase II study of tucatinib and trastuzumab for HER2-positive mCRC. Annals of Oncology*,* 33 *(*suppl_7*),* S808–S869.

[36] Vaghi, C., Mauri, G., Agostara, A. G., Patelli, G., Pizzutilo, E. G. et al. (2023). The predictive role of ERBB2 point mutations in metastatic colorectal cancer: A systematic review. Cancer Treatment Reviews*,* 112*,* 102488. 10.1016/j.ctrv.2022.102488; 36410093

[37] Hyman, D. M., Piha-Paul, S. A., Won, H., Rodon, J., Saura, C. et al. (2019). Author Correction: HER kinase inhibition in patients with HER2- and HER3-mutant cancers. Nature*,* 566 *(*7745*),* E11–E12. 10.1038/s41586-019-0974-0; 30755741

[38] Aung, K. L., Stockley, T. L., Serra, S., Kamel-Reid, S., Bedard, P. L. et al. (2016). Testing ERBB2 p.L755S kinase domain mutation as a druggable target in a patient with advanced colorectal cancer. Cold Spring Harbor Molecular Case Studies*,* 2 *(*5*),* a001016. 10.1101/mcs.a001016; 27626067 PMC5002925

[39] Guarini, C., Grassi, T., Pezzicoli, G., Porta, C. (2021). Beyond RAS and BRAF: HER2, a new actionable oncotarget in advanced colorectal cancer. International Journal of Molecular Sciences*,* 22 *(*13*),* 6813. 10.3390/ijms22136813; 34202896 PMC8268006

[40] Cen, S., Liu, Z., Pan, H., Han, W. (2021). Clinicopathologic features and treatment advances in cancers with HER2 alterations. Biochimica et Biophysica Acta. Reviews on Cancer*,* 1876 *(*2*),* 188605. 10.1016/j.bbcan.2021.188605; 34358635

[41] Meng, X., Wang, R., Huang, Z., Zhang, J., Feng, R. et al. (2014). Human epidermal growth factor receptor-2 expression in locally advanced rectal cancer: Association with response to neoadjuvant therapy and prognosis. Cancer Science*,* 105 *(*7*),* 818–824. 10.1111/cas.2014.105.issue-7 24730770 PMC4317932

[42] Connell, L. C., Mota, J. M., Braghiroli, M. I., Hoff, P. M. (2017). The rising incidence of younger patients with colorectal cancer: Questions about screening, biology, and treatment. Current Treatment Options in Oncology*,* 18 *(*4*),* 23. 10.1007/s11864-017-0463-3; 28391421

[43] Cancer Genome Atlas, N. (2012). Comprehensive molecular characterization of human colon and rectal cancer. Nature*,* 487 *(*7407*),* 330–337. 10.1038/nature11252; 22810696 PMC3401966

[44] Richman, S. D., Southward, K., Chambers, P., Cross, D., Barrett, J. et al. (2016). HER2 overexpression and amplification as a potential therapeutic target in colorectal cancer: Analysis of 3256 patients enrolled in the QUASAR, FOCUS and PICCOLO colorectal cancer trials. Journal of Pathology*,* 238 *(*4*),* 562–570. 10.1002/path.2016.238.issue-4 26690310 PMC4785607

[45] Laurent-Puig, P., Balogoun, R., Cayre, A., Le Malicot, K., Tabernero, J. et al. (2016). ERBB2 alterations a new prognostic biomarker in stage III colon cancer from a FOLFOX based adjuvant trial (PETACC8). Annals of Oncology*,* 27*,* vi151. 10.1093/annonc/mdw370.08

[46] Dong, Z., Kong, L., Wan, Z., Zhu, F., Zhong, M. et al. (2019). Somatic mutation profiling and HER2 status in KRAS-positive Chinese colorectal cancer patients. Scientific Reports*,* 9 *(*1*),* 16894. 10.1038/s41598-019-53039-y; 31729406 PMC6858340

[47] Ross, J. S., Fakih, M., Ali, S. M., Elvin, J. A., Schrock, A. B. et al. (2018). Targeting HER2 in colorectal cancer: The landscape of amplification and short variant mutations in ERBB2 and ERBB3. Cancer*,* 124 *(*7*),* 1358–1373. 10.1002/cncr.v124.7 29338072 PMC5900732

[48] Li, J. L., Lin, S. H., Chen, H. Q., Liang, L. S., Mo, X. W. et al. (2019). Clinical significance of HER2 and EGFR expression in colorectal cancer patients with ovarian metastasis. BMC Clinical Pathology*,* 19*,* 3. 10.1186/s12907-019-0085-8; 30858756 PMC6393975

[49] Bahrami, A., Khazaei, M., Hasanzadeh, M., ShahidSales, S., Joudi Mashhad, M. et al. (2018). Therapeutic potential of targeting PI3K/AKT pathway in treatment of colorectal cancer: Rational and progress. Journal of Cellular Biochemistry*,* 119 *(*3*),* 2460–2469. 10.1002/jcb.v119.3 28230287

[50] Xie, Y. H., Chen, Y. X., Fang, J. Y. (2020). Comprehensive review of targeted therapy for colorectal cancer. Signal Transduction and Targeted Therapy*,* 5 *(*1*),* 22. 10.1038/s41392-020-0116-z; 32296018 PMC7082344

[51] Mizukami, T., Izawa, N., Nakajima, T. E., Sunakawa, Y. (2019). Targeting EGFR and RAS/RAF signaling in the treatment of metastatic colorectal cancer: From current treatment strategies to future perspectives. Drugs*,* 79 *(*6*),* 633–645. 10.1007/s40265-019-01113-0; 30968289

